# Selection of a suitable photosynthetically active microalgae strain for the co-cultivation with mammalian cells

**DOI:** 10.3389/fbioe.2022.994134

**Published:** 2022-09-19

**Authors:** Sophie Dani, Johannes Windisch, Xally Montserrat Valencia Guerrero, Anne Bernhardt, Michael Gelinsky, Felix Krujatz, Anja Lode

**Affiliations:** ^1^ Centre for Translational Bone, Joint and Soft Tissue Research, Faculty of Medicine, Technische Universität Dresden, Dresden, Germany; ^2^ Institute of Natural Materials Technology, Technische Universität Dresden, Dresden, Germany

**Keywords:** bioprinting, microalgae, oxygen, tissue engineering, hydrogel, photosynthesis

## Abstract

Preventing hypoxic zones in 3D bioprinted mammalian cell-laden constructs using an internal oxygen supply could enable a more successful cultivation both *in vitro* and *in vivo*. In this study, the suitability of green microalgae as photosynthetic oxygen generators within bioprinted constructs was evaluated by defining and investigating important parameters for a successful co-culture. First, we assessed the impact of light–necessary for photosynthesis–on two non-light adapted mammalian cell types and defined red-light illumination and a temperature of 37°C as essential factors in a co-culture. The four thermotolerant microalgae strains *Chlorella sorokiniana*, *Coelastrella oocystiformis*, *Coelastrella striolata,* and *Scenedesmus* sp. were cultured both in suspension culture and 3D bioprinted constructs to assess viability and photosynthetic activity under these defined co-culture conditions. *Scenedesmus* sp. proved to be performing best under red light and 37°C as well as immobilized in a bioprinted hydrogel based on alginate. Moreover, the presence of the antibiotic ampicillin and the organic carbon-source glucose, both required for mammalian cell cultures, had no impact on bioprinted *Scenedesmus* sp. cultures regarding growth, viability, and photosynthetic activity. This study is the first to investigate the influence of mammalian cell requirements on the metabolism and photosynthetic ability of different microalgal strains. In a co-culture, the strain *Scenedesmus* sp. could provide a stable oxygenation that ensures the functionality of the mammalian cells.

## 1 Introduction

Patients with injured or diseased tissues and organs could benefit from artificial tissue substitutes. Due to an aging and expanding world population, the need for these artificial grafts is rising globally (Atala et al., 2012). The field of tissue engineering aims at restoring, repairing, or replacing functional tissues by combining biomaterials, cells and optionally biochemical or physical stimuli to create tissue substitutes suitable for clinical transplantation. One of the greatest limitations of tissue engineered constructs *in vitro* and *in vivo* is a lack or insufficiency of oxygen supply. Due to a missing or only slowly ingrowing vasculature, oxygen can reach the cells only by diffusion through the surrounding biomaterial, affecting the success of cell colonization by impeding survival and physiological function of the cells (Xiao et al., 2014). However, as the diffusion length is a restricting factor, the possible construct size is oftentimes limited to dimensions that are not clinically relevant (Lovett et al., 2009; Ke and Murphy, 2019). In search for potential solutions to this problem, the use of natural oxygen generators such as photosynthetically active microalgae or cyanobacteria has been proposed to increase oxygen levels and balance oxygen gradients in larger tissue engineered constructs.

The first application of a photosynthetically active organism as natural oxygen source was presented by Bloch et al., in 2006 who successfully co-immobilized the unicellular microalga *Chlorella sorokiniana* and pancreatic islets of Langerhans in alginate beads (Bloch et al., 2006a; Bloch et al., 2006b). There, co-immobilization of both cell types within the same beads and as well as immobilizing each cell type in separate beads led to similar results: Cultivated at 37°C in Krebs Ringer bicarbonate buffer solution under anoxic conditions over a span of 180 min, the presence of the microalgae led to higher oxygen concentrations in the solution and an increased functionality of the islets compared to a culture without microalgae. In 2014, Hopfner et al. utilized the unicellular green microalga *Chlamydomonas reinhardtii* for a direct contact co-culture with murine fibroblasts (Hopfner et al., 2014). Although the culture conditions, 30°C and a simple mix medium of mammalian cell culture and algal medium (50:50 mixture), were a compromise for both species, the expression of the hypoxia-inducible factor HIF-1α in the fibroblasts could be reduced by the presence of microalgae. Follow-up studies of this team demonstrated the biocompatibility, safety and potential applicability of microalgae-laden commercial collagen scaffolds for photosynthetic therapies in a mouse full skin defect as well as in a first clinical trial comprising eight patients with full thickness skin wounds (Schenck et al., 2015; Chávez et al., 2016; Obaíd et al., 2021).

In order to better control the exchange and interaction between the phylogenetically very different cell types in such co-cultures, our group suggested the utilization of three-dimensional (3D) bioprinting and established the immobilization of microalgae using extrusion-based 3D bioprinting (Lode et al., 2015). With this technique, cell-laden hydrogels, so-called “bioinks”, are deposited strand wise to build up layer-by-layer volumetric constructs of a defined shape and (macroporous) structure. The embedding of cells into the hydrogel-based constructs during their fabrication can implement an efficient and spatially defined colonization and thus, a patterning of different cell functionalities. That offers advantages for the creation of tissue engineered constructs close to natural conditions but also allows the separate embedding of mammalian cells and microalgae in neighboring strands, which is expected to enable an efficient oxygen transfer while reducing undesired side effects. In this first study establishing the Green Bioprinting approach, our group demonstrated that the microalgae species *C. reinhardtii* and *C. sorokiniana* can be embedded in an alginate-based 3D bioprinted construct and do not only survive the extrusion-printing process but remain viable and photosynthetically active within this translucent hydrogel construct during cultivation for 12 days (Krujatz et al., 2015; Lode et al., 2015). In addition, we demonstrated that the utilized microalgae and human cells can be combined within one scaffold in close vicinity but without direct contact by multichannel extrusion-printing (Lode et al., 2015; Trampe et al., 2018). In a very recent study, Maharjan et al. embedded bioprinted structures laden with *C. reinhardtii* in a hydrogel laden with human HepG2 cells and observed an enhanced viability and functionality of the HepG2 due to reduced hypoxic conditions over a cultivation period of 4 days (Maharjan et al., 2021).

While these studies all demonstrated the high potential of natural oxygen generators, it was also shown that many factors still need to be optimized to achieve feasible, long-term co-cultures. One important factor is photosynthetically active radiation, which is a prerequisite of algal photosynthesis but might also be toxic to naturally not light-exposed mammalian cell types. In this study, two types of non-light adapted cells were used to investigate the impact of two different wavelength ranges: 1) human primary dental pulp stem cells (DPSC) and 2) INS-1, a model cell line for insulin-producing beta cells derived from rat insulinoma (Asfari et al., 1992). DPSC were selected due to their potential to differentiate into various tissue cell types (Govindasamy et al., 2011; Staniowski et al., 2021), whereas INS-1 cells were chosen as an example for a possible functional co-culture with the potential to be further developed for the treatment of Diabetes Type I using microalgae as oxygen source. The cultivation time in this study was set to 7 days, which is significantly longer compared to previous works in this field and sufficient to evaluate the impact of the parameters adapted to the co-cultures.

Based on the demands of the mammalian cells, a suitable microalgae strain needed to be determined that can tolerate the high cell culture temperature of 37°C and remains photosynthetically active under culture conditions essential for mammalian cells. Due to their thermotolerant properties, the four unicellular microalgae strands *Chlorella sorokiniana*, *Scenedesmus* sp*.*, *Coelastrella oocystiformis* and *Coelastrella striolata* were selected to be investigated as potential oxygen generators in bioprinted constructs for this study. The microalgae’s ability to survive and stay photosynthetically active as the most important determinants were investigated under the following cultivation parameters: 1) a temperature of 37°C and an illumination regime that is not detrimental to mammalian cells (Can it provide adequate light energy for photosynthesis?), 2) the presence of glucose (Are the microalgae photosynthetically active if organic C-sources are available?), 3) the presence of common antibiotic supplements in mammalian cell culture media (Do they affect the microalgae?). As we strive for a bioprinted co-culture, the four microalgae strains were also investigated regarding their tolerance to bioprinting focusing on viability, growth, and function of the printed cultures.

## 2 Materials and methods

### 2.1 Cell expansion and experimental setup of mammalian cells

Human primary dental pulp stem cells (DPSC), isolated from deciduous teeth as previously published (Neunzehn et al., 2014), were provided by the Department of Maxillofacial Surgery at University Hospital Carl Gustav Carus Dresden. The ethics commission of TU Dresden approved the application of DPSC for experiments (EK 106042010). The rat insulinoma derived beta-cell line INS-1 was a kind donation from Prof. emeritus Claes B. Wollheim (MD Department of Cell Physiology and Metabolism, University Medical Center 1, Geneva, Switzerland) (Asfari et al., 1992). Both cell types were expanded in monolayer culture in T175 cell culture flasks (Sarstedt AG & Co. KG, Nümbrecht, Germany) at 37°C and 5% CO_2_ with twice-weekly passaging. DPSC were cultivated in Dulbecco’s modified eagle’s medium (DMEM; Gibco, Life Technologies, Germany), containing 10% fetal calf serum (FCS; Corning Inc., NY, United States), 100 U/ml penicillin and 100 μg/ml streptomycin (P/S; both Gibco, Life Technologies). INS-1 were immersed in RPMI-1640 medium with 11.1 mmol/L D-glucose (Gibco, Life Technologies) supplemented with 10% FCS, P/S, 10 mmol/L HEPES (Carl Roth GmbH + Co. KG, Germany), 2 mmol/L L-glutamine (Merck Millipore, Biochrom GmbH, and Germany), 1 mmol/L sodium pyruvate (AppliChem GmbH, and Germany), and 50 μmol/L 2-mercaptoethanol (Sigma-Aldrich, Germany). For the experiments in this study, the cells were detached from the cell culture flasks, and 1.3 × 10^5^ cells/cm^2^ (INS-1) or 1 × 10^4^ cells/cm^2^ (DPSC) were seeded into the wells of a six-well tissue culture plate (Corning Inc.). Exposure of mammalian cells to photosynthetically active radiation was performed by placing the tissue culture plates on top of an LED light panel (Axis GmbH & Co. KG, Nürnberg, Germany) to ensure homogeneous illumination with either 20 µmol/m^2^s white light (400–700 nm) or 10 µmol/m^2^s red light (600–650 nm). The entire experimental setup was placed in a CO_2_ incubator (Heracell, Thermo Fisher Scientific Inc., United States). Cultivation was performed for 7 days at 37°C with change of medium at day 4.

### 2.2 Cell expansion and experimental setup of microalgae in suspension culture

*Chlorella sorokiniana* SAG 211-8k, *Coelastrella striolata* SAG 16.95 and *Coelastrella oocystiformis* SAG 277-1 were obtained from the Collection of Algae at Goettingen University (Germany), *Scenedesmus* sp. CCALA 1074 was purchased from the Culture Collection of Autotrophic Organisms (CCALA) (Třeboň, Czec Republic). The microalgal strains were inoculated in 50 ml modified tris-phosphate (TP) medium (according to (Harris, 1989)); the ammonium chloride (NH_4_Cl; Merck KGaA, Germany) originally present in the medium was substituted with 0.75 g/L sodium nitrate (NaNO_3_, Merck KGaA). Cell expansion was performed in 250 ml Erlenmeyer shake flasks (ROTILABO®, Carl Roth GmbH + Co. KG) at 25°C for 7 days, followed by sub-cultivation of 5 ml into 45 ml fresh TP medium. For experiments with suspension cultures described in this study, cultures were inoculated at an OD_750_ of 0.1 in the respective medium (TP medium or TP medium supplemented with P/S or with 100 U/ml penicillin) and cultivated at 26°C up to 7 days without change of medium. They were illuminated constantly with white or red LED light (35 µmol/m^2^s average intensity, white light 400–700 nm, red light 600–650 nm) using a light plate on the ceiling of the incubator (Memmert IPP200, Memmert GmbH + Co. KG, Germany) allowing a uniform illumination of the shake flask.

### 2.3 Bioprinting and experimental setup of microalgae-laden constructs

Extrusion-based 3D printing, also called 3D plotting, was used for bioprinting of the algae-laden constructs as described previously (Krujatz et al., 2015; Lode et al., 2015). To prepare the biomaterial ink, alginic acid sodium salt from brown algae (alg; M/G ratio 1:2, Sigma-Aldrich) was dissolved in deionized water at a concentration of 30 mg/ml by stirring overnight. After sterilization of the solution by autoclaving at 121°C for 20 min, 90 mg/ml methylcellulose (MC; M0512, molecular weight ≈88 kDa, 4.000 cPs, Sigma-Aldrich), autoclaved in powder form at 121°C for 20 min, was carefully mixed into the alginate solution before letting the material rest for 90 min to allow the MC to swell. Subsequently, microalgae were mixed into the biomaterial ink in a concentration of 1 × 10^6^ cells, suspended in 100 μl TP medium, per Gram biomaterial ink. The resulting bioink was transferred into a 10 ml plotting cartridge (Nordson EFD, Oberhaching, Germany) and plotted by a pneumatic-driven extrusion printer (Bioscaffolder 3.1; GeSiM mbH, Radeberg, Germany). The bioinks were extruded through dosing needles (d = 410 μm; Globaco, Rödermark, Germany) with 200 kPa air pressure, a plotting speed of 6 mm/s and a layer height of 0.27 mm. Cuboidal scaffolds (base area 16 × 16 mm^2^) with a strand distance of 3 mm, five layers and a layer-to-layer orientation of 90° were fabricated. Immediately after printing, the scaffolds were ionically crosslinked for 10 min using Ca^2+^-ions in a 100 mM CaCl_2_ solution and subsequently cultured in 12-well tissue culture plates (Corning Inc.), submerged in 2 ml of respective medium (TP medium or TP medium supplemented with P/S, with 100 U/mL penicillin, with 100 μg/ml ampicillin (Carl Roth GmbH + Co. KG) or with 7.1 mM glucose). The bioprinted constructs were continuously illuminated with red light (600–650 nm; PAR of 150 µmol/m^2^s) from the bottom using an RGBW LED strip (LS LED Lighting, Amsterdam, Netherlands), installed in the incubator (Heracell, Thermo Fisher Scientific Inc.). Cultivation was performed for 7 days at 37°C with change of medium at day 4.

### 2.4 Assessment of cell viability

The cell viability of mammalian cells was investigated using simultaneous live/dead staining with Calcein-AM/ethidium homodimer-1 (LIVE/DEAD Viability/Cytotoxicity Kit for mammalian cells, Thermo Fisher Scientific, United States) following the manufacturer’s protocol. Fluorescence microscopy using a BZ-X800 fluorescent light microscope (Keyence Corporation, Japan) followed by semi-automatic area determination of living and dead cells using ImageJ (Fiji, Version 1.52p) (Schindelin et al., 2012) was used to assess the quantitative cell viability. Viability was defined as the ratio of the area of living cells divided by the sum of areas of live and dead cells. Viability of bioprinted microalgae was assessed by staining dead cells with SYTOX™ Green Nucleic Acid Stain (Thermo Fisher Scientific, United States) and exploiting chlorophyll autofluorescence for visualization of live cells as described previously (Lode et al., 2015).

Cell viability of microalgal suspension cultures was quantified by flow cytometry. Prior to analysis, cells were diluted using physiological saline solution (0.9% NaCl) to an optical density of OD_750_ = 0.1, followed by staining at a working concentration of 10 µM SYTOX™ Green Nucleic Acid Stain (Thermo Fisher Scientific Inc.) and an incubation time of 20 min at room temperature. The flow cytometry analysis was performed using a CyFlow Cube 8 flow cytometer (Sysmex Europe GmbH, Norderstedt, Germany) equipped with a 488 nm solid state laser and four fluorescence channels. For quantification, the “cells in region” protocol of the Cube 8 was used; the measurement was terminated and evaluated after the analysis of 50,000 cells (FCS Express, DeNovo Software, CA, United States).

### 2.5 Measurement of cell growth

To analyze the growth of bioprinted microalgae, the chlorophyll content was determined and correlated with the cell concentration. For sampling, whole bioprinted constructs were stored at −80°C until further analysis. After thawing, 3 ml of 100 mM sodium citrate solution were added to dissolve the constructs at 4°C overnight. Dissolved samples were centrifuged at 12,000 rpm for 15 min and the supernatant was removed. The microalgal pellet was resuspended in 250 μl dimethyl sulphoxide (DMSO) and transferred to a Precelllys® tube (Peqlab, Erlangen, Germany). The suspension was frozen at −80°C for 20 min, and after thawing, 1 ml of DMSO and three ceramic Precellys® beads (Peqlab) were added. In the cell homogeniser (Precelllys® 24 system, Peqlab), the tube was shaken three times for 30 s at 5,000 rpm. 100 µl of lysate were transferred to a transparent 96-well plate and the optical density was measured at 435 nm using a microplate reader (Infinite M200 pro, Tecan Trading AG, Switzerland).

The concentration of the mammalian cells at different time points of cultivation was determined by quantification of the DNA content after cell lysis which was correlated with a calibration line obtained from defined cell numbers. Cell-seeded well plates, taken at different time points of cultivation, were frozen at −80°C until analysis. After thawing, 3 ml of 100 mM sodium citrate solution were added to each well and the sealed plates were incubated at 60°C overnight for cell lysis, followed by 10 min sonication on ice. DNA quantification in the lysates was performed using the QuantiFluor dsDNA system (Promega Corporation, United States) as per the manufacturer’s instructions; Relative Fluorescence Units were measured at excitation and emission wavelengths of 485 and 535 nm, respectively, using the microplate reader (Infinite M200 pro, Tecan).

### 2.6 Oxygen measurement

The optical oxygen measurement device “Resipher” (Lucid Scientific, Atlanta, GA, United States) was used for all oxygen concentration measurements. Bioprinted constructs were cultivated in a 96-well plate with 150 µl of either TP medium or TP medium supplemented with 7.1 mM of glucose, while the oxygen content was measured continuously over the course of the experiment. At the end of the experiment, samples and supernatant were stored at −80°C and −20°C, respectively, before analyzing the glucose content *via* DNS assay (Section 2.7) and cell concentration *via* analysis of the chlorophyll content (Section 2.5).

### 2.7 Glucose measurement

Using 3,5-Dinitrosalicylic acid (DNS), the glucose concentration of the supernatant was determined as a reducing sugar using a colorimetric process. 100 µl sample volume was mixed in a ratio of 1:1 with DNS-reagent and heated in a water bath at 99°C. After diluting the sample with double distilled water in a ratio of 1:4, the extinction of the solution was analyzed using the microplate reader (Infinite M200 pro, Tecan) at a wavelength of 540 nm.

### 2.8 Evaluation of photosynthetic activity

The photosynthetic efficiency of microalgae (suspended and bioprinted) was determined using pulse-amplitude modulated fluorometry (PAM) utilizing the stationary measurement device MINI-PAM blue (Heinz Walz GmbH, Effeltrich, Germany, equipped with a blue LED), and the control software ImagingWinGigE (Heinz Walz GmbH). The blue LED emitted a beam of low-intensity actinic light that produced a minimal fluorescent yield due to open reaction centers in the photosystem II (F_0_), followed by a saturation beam of higher intensity, that caused the reaction centers to close as a protective reaction, producing a maximal fluorescence yield (F_M_). To calibrate the measurements, the ratio F_0_/F_M_ was determined using the standardized function of the software. In order to obtain information about the photosynthetic performance of the microalgae, induction kinetics were recorded by applying saturation light pulses to the samples repeatedly in 20 s intervals during actinic illumination. The fluorescent radiation emitted in response to both beams was set in proportion and was used to calculate the Effective Photosystem II Quantum yield (Y(II), photosynthetic activity). Y(II) illustrates the efficiency of quantum utilization in photosystem II (PS II), a higher Y(II) value signals that a higher percentage of the photons absorbed by PS II have been converted into chemically fixed energy usable by the cell. Induction measurements were taken over 300 s at optimal intensity, all samples were adapted to the dark for at least 5 minutes to open all reaction centers. For comparison of photosynthetic activity within this study, the recorded Y(II) values under saturation light were averaged over a measurement and depicted as bar graphs instead of presenting the complete induction kinetic curve. Light curves were recorded in increasing light intensities of 1, 6, 48, 124, 278, 427, 626, and 962 µmol/m^2^s; ahead of light curve measurements, the cells were adapted to ambient light. For suspensions cultures, 250 µl of culture were placed in a Petri dish under the imaging head of the device, the Area of Interest (AoI) was set to the diameter of the dish. To account for the macroporous structure of bioprinted constructs, five smaller AoI were set for each sample on defined positions and averaged for calculation.

### 2.9 Statistical analysis

All results were evaluated by one-way Analysis of Variance (ANOVA), followed by Tukey’s multiple comparison test with GraphPad Prism 7 software. Significant differences were assumed at *p < 0.05; **p < 0.01; ***p < 0.001.

## 3 Results

### 3.1 Influence of white and red light on viability and growth of mammalian cells

**FIGURE 1:**
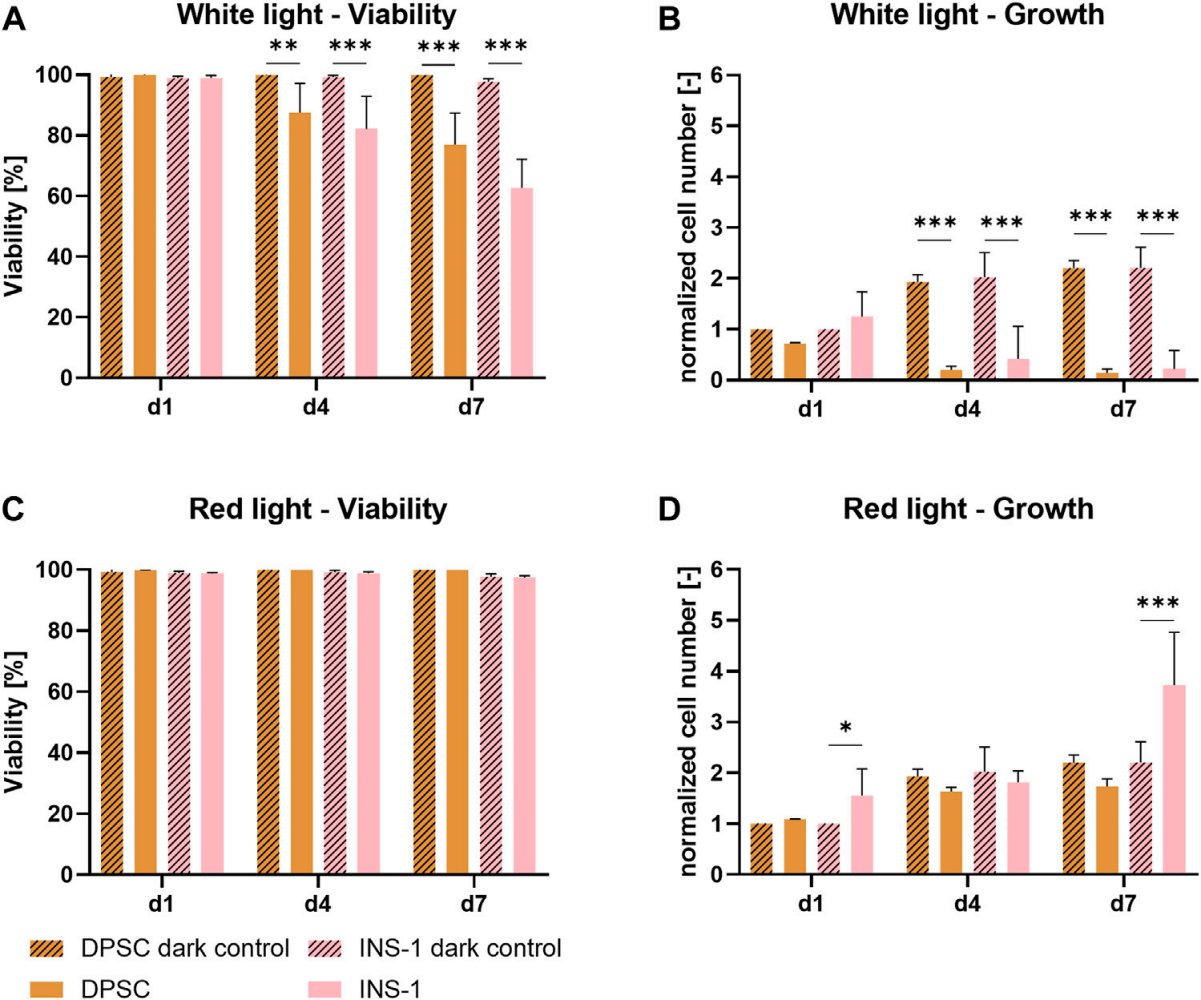
Viability **(A,C)** and growth **(B,D)** of DPSC and INS-1 monolayer cultures, cultivated over 7 days with white- or red-light illumination; usual cultivation in the dark was conducted as control. The cell numbers **(B,D)** were normalized to the cell number of the respective dark control group on day one. Depicted are mean ± standard deviation, n ≥ 12 **(A,C)**, n ≥ 3 **(B,D)**; *p < 0.05; ***p < 0.001.

Figures 1A,B show the influence of white light on DPSC and INS-1. Cell concentrations obtained from DNA quantification were normalized to the cell concentration of the dark control group on day one to illustrate relative growth. A significant decrease in viability and cell number is demonstrated for both cell types under illumination after four and 7 days of culture in comparison to the control cultures in the dark, indicating an adverse effect of white light on the cells. The viability of DPSC decreased from 98% on day one to 76% after 7 days of illumination; this effect was observed even more strongly for INS-1, with only 63% metabolically active cells on day seven. The relative cell number of both control groups in darkness stagnated after an approx. 2-fold increase by day four, presumably because the whole area in the well was covered by cells. Under illumination with white light, the cell number is significantly reduced over time, with a strong decrease between day one and day four and barely any cells left on day seven.

In contrast, as shown in Figures 1C,D, there is no detrimental effect of red light on either DPSC or INS-1. The viability of all groups ranged between 98 % and 99% over the time period of the experiment, cell numbers under illumination showed a similar behavior to their respective non-illuminated counterparts. Notable is a significantly higher relative cell number of illuminated INS-1 on day seven compared to the dark control group.

### 3.2 Screening of four thermotolerant microalgae strains to identify a suitable co-culture partner

#### 3.2.1 Photosynthetic efficiency of the selected microalgae strains under optimal and mammalian-cell compatible culture environments

**FIGURE 2:**
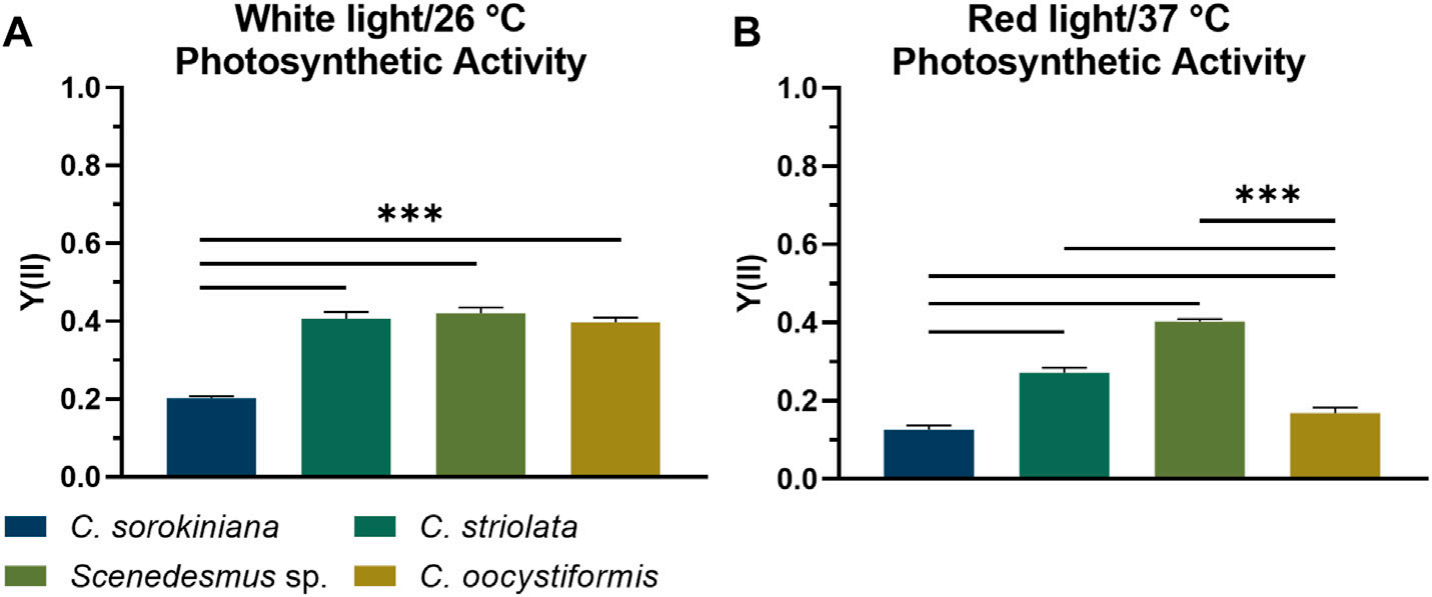
PAM induction curve measurement; averaged saturated effective photosynthetic yield Y(II) of the four microalgae strains in suspension culture under either white light illumination at 26°C **(A)** or red-light illumination at 37°C **(B)** after 3 days of cultivation in TP medium. Depicted are mean ± standard deviation, n = 5; ***p < 0.001.

Pulse-Amplitude Modulated Fluorometry (PAM) was used to determine the photosynthetic activity of microalgae in suspension and immobilized cultures, based on the principles of chlorophyll fluorescence as described elsewhere (Masojídek et al., 2004). By applying PAM induction kinetic measurements, as described in 2.8, the photosynthetic efficiency of the selected microalgae strains *Scenedesmus* sp., *Chlorella sorokiniana*, *Coelastrella striolata, and Coelastrella oocystiformis* was investigated in suspension culture under the temperature and light conditions imposed by the mammalian cells (red light illumination at 37°C) in comparison to the optimal strain maintenance conditions (white light illumination at 26°C). After cultivation for 3 days under optimal culture conditions, the three strains *C. striolata*, *C. oocystiformis*, and *Scenedesmus* sp. all achieved the same level of photosynthetic activity, with values of approx. Y(II) = 0.4; the strain *C. sorokiniana* reached values of only Y(II) = 0.2 (Figure 2A). After 3 days of cultivation in mammalian cell-compatible conditions, Y(II) of *C. sorokiniana* averaged only 0.127, *C. oocystiformis* achieved 0.169, surpassed by *C. striolata* with values of 0.272 and *Scenedesmus* sp., with 0.403 (Figure 2B). Thus, *Scendedesmus* sp. was the only strain achieving comparable photosynthetic efficiency in optimal and mammalian-cell-adapted conditions.

**FIGURE 3:**
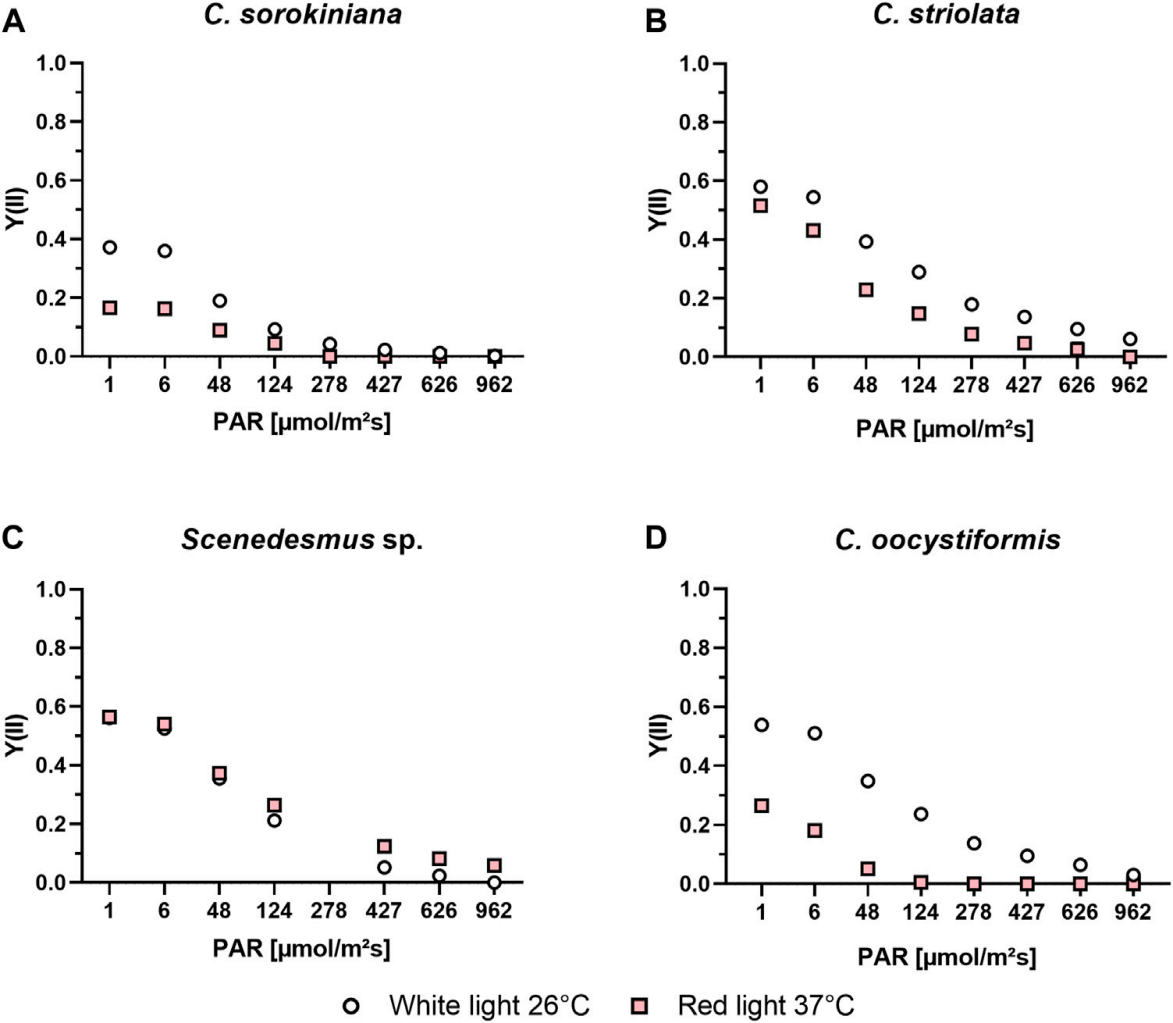
PAM light curve measurements of *C. sorokiniana* **(A)**, *C. striolata* **(B)**, *Scenedesmus* sp. **(C)** and *C. oocystiformis* **(D)** in suspension culture under both optimal (white light and 26°C) and mammalian cell-compatible (red light and 37°C) conditions in TP medium.

The light curve progressions (Figure 3) of the microalgae suspensions cultivated under optimal conditions (white light at 26°C) displayed similar relationships between the strains as already seen when investigating the induction kinetics. In all cases photosynthetic activity was highest at lower intensities, with a decrease observed at ≥ 48 µmol/m^2^s, but the level of activity, especially at the highest concetrations varied between the strains. *C. sorokiniana* showed the lowest level of activity overall, and no photosynthetic acitivity could be detected at intensities of 427 and higher. The other strains showed a higher starting activity of around Y = 0.6, which was followed by a nearly as rapid drop in activity at higher intensities though. The corresponding Y(II) values measured for the suspensions cultivated under red light at 37°C revealed an even lower photosynthetic activity of *C. sorokiniana* in these conditions; at low intensities, values around Y(II) = 0.2 were achieved while no activity could be detected at a light intensity of 278 µmol/m^2^s or higher. The activity for *C. oocystiformis* was also clearly reduced under red light at 37 °C in comparison to the optimal condition, with no Y(II) measurable at 124 µmol/m^2^s and higher. *C. striolata* also showed lower Y(II) values under red light at 37°C, but the differences from culture under white light at 26°C were markedly less pronounced than in the two strains described above. The most noteworthy result was obtained for *Scenedesmus* sp. here, the sample cultivated under red light at 37°C achieved similar Y(II) values at low light intensities and remarkably even higher Y(II) values at higher intensities compared to the culture grown under white light at 26°C.

#### 3.1.2 Suitability of the selected microalgae strains for bioprinting

**FIGURE 4:**
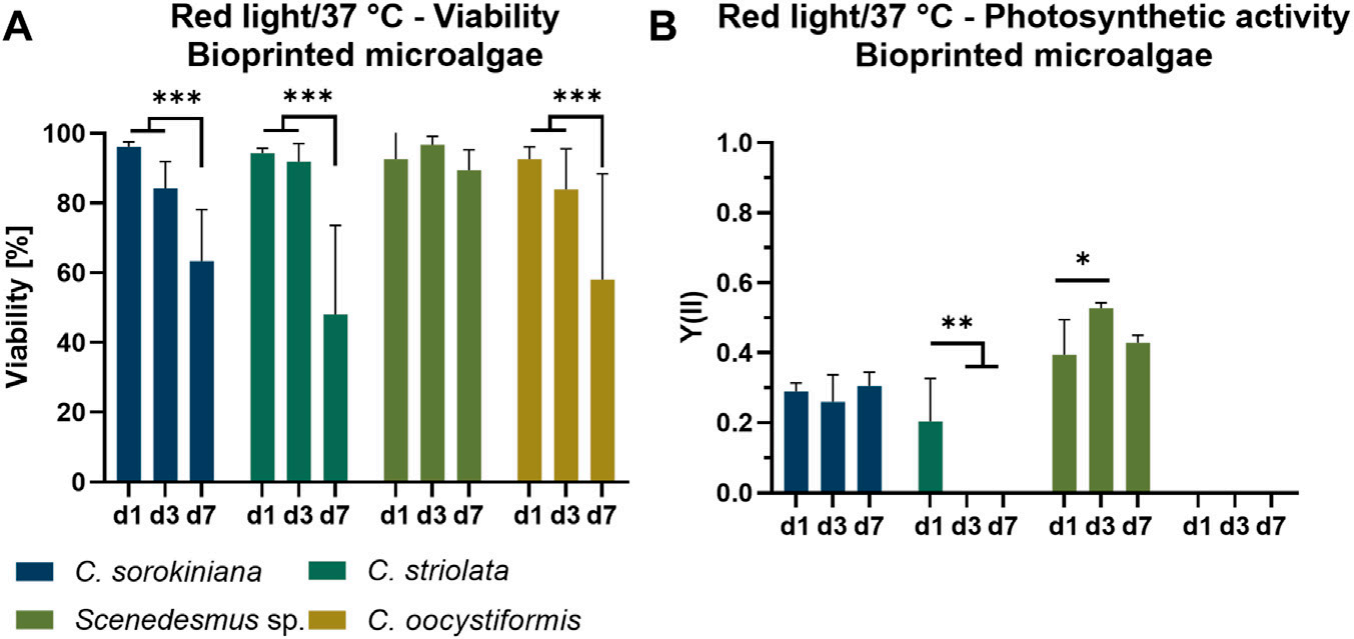
Viability **(A)** and photosynthetic activity **(B)** of 3D bioprinted microalgae, cultivated in TP medium over the span of 7 days under red light illumination (150 µmol/m^2^s) at 37°C. Depicted are mean ± standard deviation, n = 6; *p < 0.05; **p < 0.01¸ ***p < 0.001.

To investigate the compatibility of the four microalgae strains with the process of extrusion-based bioprinting as well as with the resulting immobilization in the alginate-based hydrogel matrix, the viability and photosynthetic efficiency of the bioprinted microalgae were monitored during cultivation in TP medium under mammalian cell-compatible conditions (red light, 37°C) for 7 days (Figure 4). Images of the resulting constructs of microalgae embedded in the translucent ink are shown in Supplementary Figure S1. On day one of culture, viability of all strains was consistently above 90%, indicating a high tolerance of the microalgae towards the extrusion process. Immobilization on the other hand had a detrimental effect on three of the four strains: Viability dropped to between 50% and 60% over the course of 7 days, with a constant decrease in *C. sorokiniana* and *C. oocystiformis* constructs. In *C. striolata* constructs, this effect seemed to be delayed as the viability was still around 90% on day three and decreased subsequently until day seven. In contrast, the results of the strain *Scenedesmus* sp. indicated a constantly high viability of >90% over the whole culture period (Figure 4A).

The photosynthetic yield Y(II) measured for the microalgae-loaded scaffolds is shown in Figure 4B. Of the four immobilized microalgae strains, only two showed photosynthetic activity over the entire time of observation: *C. sorokiniana* achieved relatively constant values of approximately Y(II) = 0.25–0.3 over 7 days; a similar behavior was observed for *Scenedesmus* sp. but with higher values of approximately Y(II) = 0.4–0.5. *C. striolata* showed photosynthetic activity only at day one, shortly after the printing process, but the effectiveness is below those of *C. sorokiniana* and *Scenedesmus* sp., with values around Y(II) = 0.2. In the case of *C. oocystiformis*, no photosynthetic activity could be measured at any time point.

The screening of the four thermophilic microalgae strains identified *Scenedesmus* sp. as suitable partner for the co-culture with mammalian cells in a bioprinting approach as this strain was demonstrated to be compatible 1) with culture conditions adapted to the needs of mammalian cells (red light, 37°C)—without any reduction in viability and photosynthetic activity–and 2) with bioprinting. Therefore, the study was continued with *Scenedesmus* sp. cultures.

### 3.3 Influence of antibiotics on viability and photosynthetic efficiency of *Scenedesmus* sp.

**FIGURE 5:**
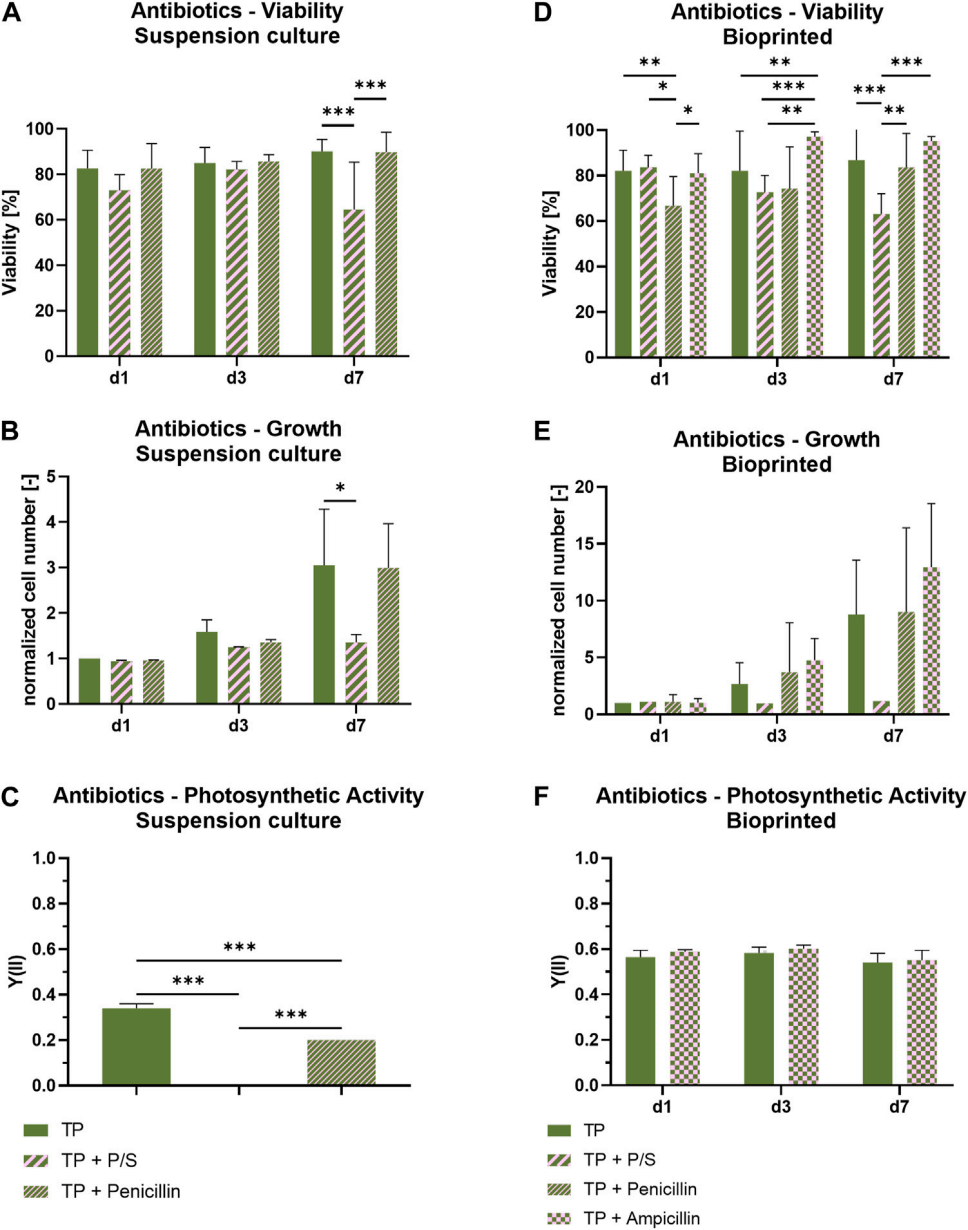
Viability, cell growth and photosynthetic activity of suspension cultures **(A–C)** and 3D bioprinted cultures **(D–F)** of *Scenedesmus* sp. cultivated over 7 days under red illumination (150 µmol/m^2^s) at 37°C in TP medium without and with antibiotics (P/S: 100 U/ml penicillin and 100 μg/ml streptomycin; 100 U/ml penicillin; 100 μg/ml ampicillin). The cell numbers **(B,E)** were normalized to the cell number measured on day one for the respective group. Depicted are mean ± standard deviation, n = 6¸ *p < 0.05; **p < 0.01¸ ***p < 0.001.

To prevent bacterial contamination, most mammalian cell culture media are supplemented with antibiotics; due to the many handling steps necessary in the fabrication of bioprinted constructs, antibiotic supplements gain an even bigger importance in this context. In order to evaluate the compatibility of antibiotics with *Scenedesmus* sp., penicillin and streptomycin as a mixture (P/S) and penicillin alone were added to suspension cultures of *Scenedesmus* sp. in TP medium and their influence on viability and growth was investigated (Figures 5A–C). While there was no effect of the antibiotics on day one and day three of cultivation, a decrease in viability by 20% as well as a stagnating cell number were noticed on day seven in P/S supplemented TP medium compared to the antibiotic-free medium and no photosynthetic activity was detectable at this time point. In contrast, the cultivation of microalgae in TP medium supplemented with only penicillin resulted in only a slight decrease in the Y(II) while viability and growth remained unaffected. It can be concluded that streptomycin has a negative impact on photosynthetic activity and growth of the microalgae. While penicillin did not influence the microalgae’s viability or function, its working mechanism is limited to Gram-positive bacteria and only some Gram-negative cocci. Therefore, ampicillin, a semisynthetic antibiotic derived from penicillin that affects Gram-positive as well as Gram-negative bacteria, was investigated as a possible replacement for the penicillin/streptomycin combination. Since its mechanism of action is similar to penicillin, ampicillin was expected to be compatible with the microalgae. In order to additionally evaluate whether the biomaterial ink could (at least partially) protect the microalgae from the antibacterial supplements, the subsequent experiment was carried out using bioprinted *Scenedesmus* sp. cultures. In this setup, the effect of working concentrations of P/S, penicillin, and ampicillin on viability and growth of the microalgae were investigated over the course of 7 days (Figures 5D–F). While P/S exhibited the same adverse effects observed in suspension cultures with lowered viability rates and cell numbers, no negative effect was detectable in TP medium supplemented with ampicillin. Penicillin-supplemented medium resulted in a reduced viability of 75%, similar to P/S-containing medium, on day four; however, on day seven, it recovered to 90% viable cells, the same level of viability as observed for antibiotic-free medium and medium supplemented with ampicillin. The cell number development was not impacted by the low viability on day four; it remained comparable to the antibiotic-free medium at all times. Since the addition of ampicillin did not affect the microalgae’s viability or growth rate, it was a promising candidate to replace penicillin/streptomycin in a co-culture medium. To confirm this finding, bioprinted cultures of *Scenedesmus* sp. cultivated in either antibiotic-free or ampicillin-containing medium were analyzed regarding their photosynthetic activity over the course of 7 days. At all time points, the Y(II) of both groups were at a comparable, high level of 0.5–0.6, proving that ampicillin has no adverse effect on the function and growth of *Scenedesmus* sp. (Figure 5F).

### 3.4 Influence of glucose on viability, photosynthetic efficiency, and oxygen production of *Scenedesmus* sp.

**FIGURE 6:**
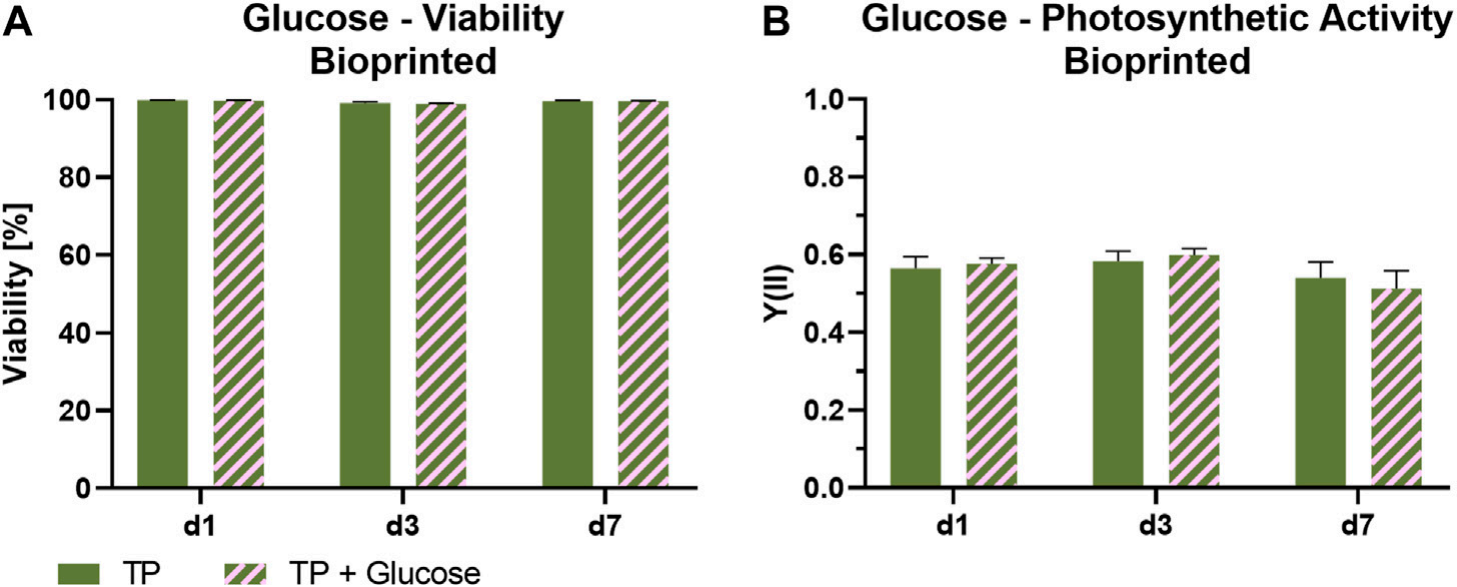
Viability **(A)** and photosynthetic activity **(B)** of 3D bioprinted *Scenedesmus* sp.; cultivated over 7 days under red illumination (150 µmol/m^2^s) at 37°C in TP medium without and with 7.1 mM glucose. Depicted are mean ± standard deviation, n ≥ 12 **(A)**, n ≥ 3 **(B)**.

**FIGURE 7:**
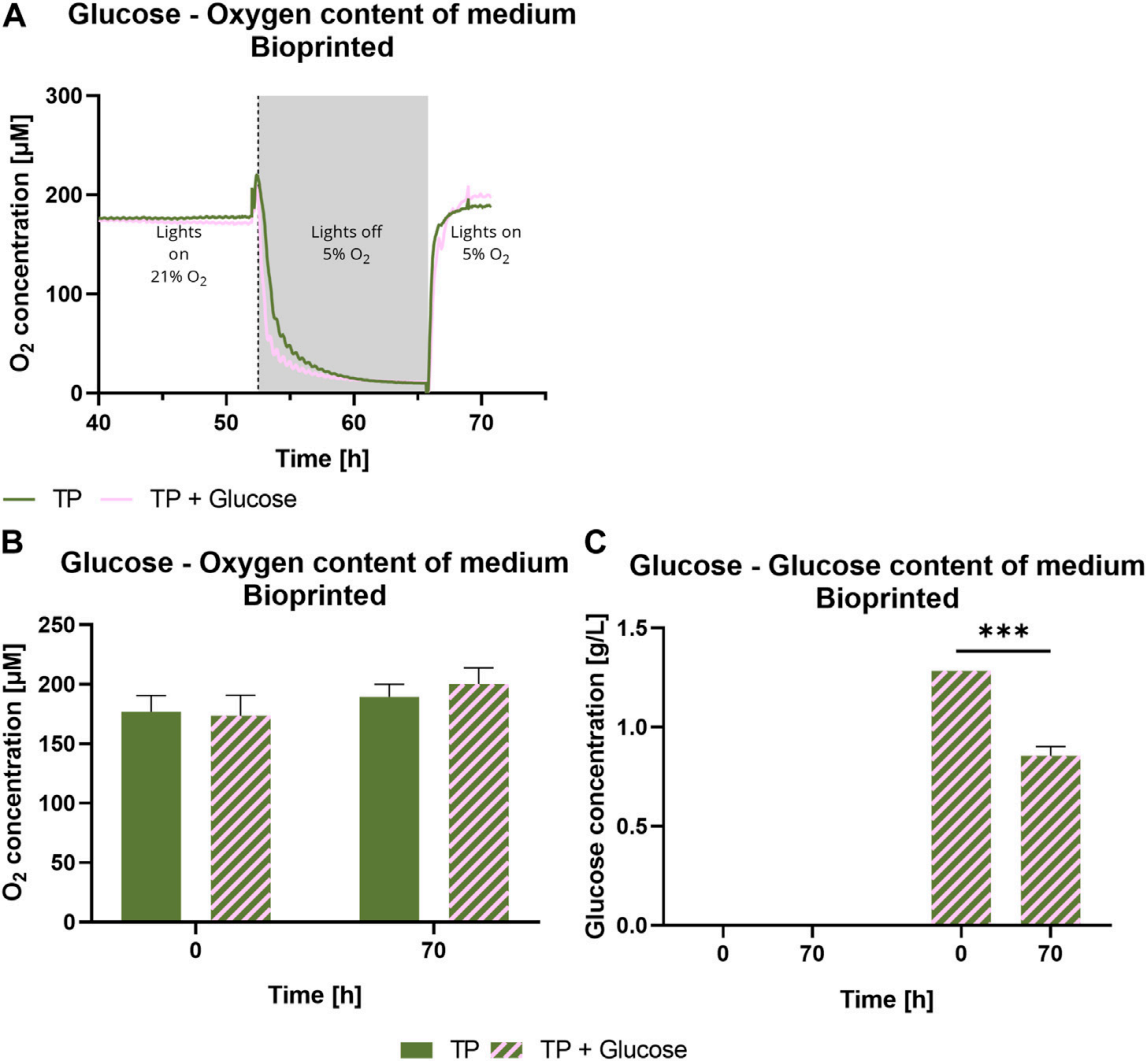
Oxygen content over time **(A)**, at 0 and 70 h **(B)** and glucose concentration **(C)** of medium of 3D bioprinted *Scenedesmus* sp.; cultivated for 3 days under red illumination (150 µmol/m^2^s) at 37°C and normoxia in TP medium without and with 7.1 mM glucose before the oxygen content was decreased to 5% after approximately 52 h. Simultaneously, the light was turned off until the samples reached a hypoxic state. Depicted are mean values, n = 4 **(A)**, mean ± standard deviation, *n* = 4 **(B,C)**; ***p < 0.001.

To investigate the impact of the presence of glucose on the photosynthetic activity, bioprinted *Scenedesmus* sp. was cultivated under mammalian cell-compatible conditions either in the standard TP medium or in TP medium containing 7.1 mM glucose, which is in the range of glucose concentrations in typical mammalian cell culture media. Figure 6 depicts the influence of glucose on viability and photosynthetic activity of *Scenedesmus* sp. Over the course of 7 days, the presence of glucose showed no effect on the microalgae for either aspect. While the viability rate for both glucose-containing and glucose-free medium was above 98% at all time points, the unchanged high photosynthetic yield in the presence of glucose is particularly remarkable. The ratio of photoautotrophic and chemoheterotrophic metabolism in the presence of glucose was further evaluated by measurement of the development of oxygen and glucose concentration in the culture medium (Figure 7). For this purpose, the bioprinted microalgae were cultivated for 2 days in normoxic conditions and under red light illumination at 37°C. On day two, the medium was refreshed before the oxygen concentration within the incubator was set to 5%; simultaneously, the light was turned off to inhibit photosynthetic activity in order to deplete potential oxygen production from the bioprinted constructs. On day three, the light was turned on again to observe possible differences in the oxygen production of the microalgae depending on glucose availability. The oxygen concentration in both media–TP and TP + glucose–showed a similar trend: the slope of both the oxygen decrease at the start of the dark phase as well as of the oxygen increase after illumination was restored was identical, also reaching similar oxygen levels of 190 µM (Figures 7A,B). Figure 7C displays the glucose concentration in both media at the start of both experiments and after the oxygen measurement was carried out. Here, a significant decline from 1.2 g/L to 0.8 g/L can be observed. The similar development of oxygen concentration for both media and high concentrations of glucose after cultivation as well as a high photosynthetic activity over the course of 7 days, reveals that the photosynthetic oxygen production of *Scenedesmus* sp. is not reduced in the presence of the organic carbon source glucose.

## 4 Discussion

Light is essential to enable photosynthetic activity of the microalgae. Some mammalian cells, for example keratinocytes, are adapted to illumination; the vast majority of cells however are naturally not exposed to light. In the literature, several studies reported on investigation of the effect of so-called photobiomodulation, the influence a short exposure of light has on non-light adapted mammalian cells. However, the periods of illumination are kept in the range of fractions of a second up to a few minutes over the span of several days. The effect of continuous illumination on mammalian cells was not yet described. Red light emitted from lasers or LEDs is the most commonly used illumination regime for photobiomodulation, while microalgae are usually exposed to warm white light imitating sunlight (Huang et al., 2009; George et al., 2018). Red and white light were therefore investigated regarding their influence on both mammalian cell types in this study. A significant decrease in cell number and cell viability was found for both cell types when illuminated with white light. Contrary, red light illumination proved to have no negative influence on either cell type over the span of 7 days (Figure 1). These findings indicate that the wavelength has a significant impact on the effect of continuous illumination on non-light adapted cells: low-energetic red light provides a viable environment for the cells while white light, containing a portion of high-energetic blue light, has a detrimental effect on both viability and growth. The literature supports this hypothesis, with several studies showing positive effects of short bursts of red LED light on proliferation and specific marker expression of human cell types, while green and blue light were associated with inhibited cell growth and cell maturation after only seconds of exposure (Wang et al., 2017; Tani et al., 2018; Winter et al., 2018; Rosenberg et al., 2020). With these observations, red light illumination and 37°C were defined as physical culture parameters for the envisaged co-cultures of mammalian cells and microalgae.

The ability of the mammalian cell-compatible culture conditions (red light, 37°C) to support photosynthesis of the microalgae was analyzed by PAM measurements determining the photosynthetic efficiency Y(II) in comparison to the optimal culture conditions (white light, 26°C). Under optimal conditions, the Y(II) value of *C. sorokiniana* was significantly lower than those of *C. striolata*, *C. oocystiformis* and *Scenedesmus* sp. (Figure 2A), however, it is consistent with the measurements of Liu et al., who also analyzed suspension cultures of *C. sorokiniana* in TP medium (Liu et al., 2012). A change to the mammalian cell-compatible conditions led to significant Y(II) losses in three of the four strains. *C. sorokiniana*, *C. striolata* and *C. oocystiformis* all showed a clear drop in photosynthetic efficiency under red light and at 37°C compared to their respective yields under optimal conditions. In contrast, the strain *Scenedesmus* sp. seems to lose hardly any photosynthetic efficiency, as the measured Y(II) remained almost at the average level of the white light/26°C culture (Figure 2B).

Limiting the wavelength of light available to microalgae has been described as negatively impacting the photosynthetic activity in the literature. De Mooij et al. investigated the influence of different light wavelengths on Y(II) and biomass production by the microalga *Chlamydomonas reinhardtii* at 25°C (de Mooij et al., 2016). They found a significantly lower biomass production when cultivated in red light compared to cultivation in sunlight, yellow light, and warm white light: For example, Y(II) values of *C. reinhardtii* decreased from 0.64 in white light to 0.53 in red light. This is consistent with the decreases observed in *C. sorokiniana*, *C. striolata*, and *C. oocystiformis* in this study. Since chlorophyll a possesses absorption maxima in the blue and the red light range, white light is transformed into energy most efficiently in the photosynthetic process. Nevertheless, *Scendesmus* sp. and *C. sorokiniana* were reported to produce oxygen and biomass in relevant quantities under red light illumination (Mattos et al., 2015; Lee et al., 2019; Raeisossadati et al., 2020). Sub-optimal temperatures are linked to a reduced carbon metabolism of microalgal cultures due to increased photoinhibition (Bongi and Long, 1987; Sukenik et al., 1987; Vonshak and Torzillo, 2004). The negative effect of increasing temperature on the efficiency of photosystem II has been documented for example by Claquin et al. who showed a very strong decrease in photosynthetic efficiency when exceeding a critical temperature for the microalgae, using eight different marine microalgae as examples (Claquin et al., 2008). Additionally, Schreiber et al. demonstrated a heat induced limitation of the photosynthetic activity in plant cells, which follow comparable metabolic processes and adaptions to stress as microalgae (Vonshak and Torzillo, 2004; Schreiber and Klughammer, 2008). All four strains investigated in the present study were selected for their thermotolerant behavior, nevertheless, an efficiency-reducing effect of the increased temperature, especially under the discussed non-optimal light conditions, cannot be ruled out.

Even if the photosynthetic yield of *Scenedesmus* sp. under mammalian cell-compatible conditions is a good indicator for the efficiency of the photosynthesis process, induction kinetics cannot determine with certainty whether all the processes following the quantum absorption also proceed with the same effectiveness. This is based on the fact that the quantum absorption, happening in the antenna complexes of the photosynthetic apparatus, does not immediately react to environmental stress, instead, enzymatic reactions of the Calvin cycle regulate the electron turnover through PS II in a matter of milliseconds (Sukenik et al., 1987). As described by Schreiber et al., cell stress reduces the threshold at which the PS II of a cell reacts sensitively to light exposure, indicated by a significant drop of its Y(II) values. Therefore, in the course of a light curve recording under both the optimal (white light at 26°C) and the adapted (red light at 37°C) conditions, the light intensity was stepwise increased on suspension cultures until the light intensity was too high for the cells to still be photosynthetically active (Figure 3). Comparing the results of the light curve measurements of the white-light and red-light cultures of the strains at the respective temperatures, the results expected after examining the induction curves were seen. The recorded light curves indicate an increased stress level of the cells under red light compared to optimal conditions, by the losses shown. Also, the recorded light curves of *Scenedesmus* sp. seem to confirm the investigation result that the change from white to red light as well as from a temperature of 26–37°C has no negative effect on photosynthetic efficiency and stress level of the cells (Schreiber and Klughammer, 2008). In conclusion, *C. sorokiniana*, *C. striolata* and *C. oocystiformis* show varying levels of environmental stress in red light and 37°C conditions, while *Scenedesmus* sp. does not appear to be influenced. Deeper analyses regarding the metabolic processes and the state of photosynthetic units would be beneficial since the reason for these differences is not yet known.

Concluding from the high viability measured on day one (Figure 4A), the printing as well as the subsequent crosslinking process with 100 mM CaCl_2_ do not seem to have a negative impact on the cells of any of the four microalgae strains. This is in line with previous observations for the printing of the strain *C. sorokiniana* and *C. reinhardtii* in alginate-based hydrogels (Krujatz et al., 2015; Lode et al., 2015; Trampe et al., 2018; Maharjan et al., 2021). Thus, in principle, the strains all appear to be suitable for undergoing the printing process without significant cell damage. While all strains show a high viability directly after the printing process, the development of the living portion differed on the following measurement days: *Scenedesmus* sp. maintained a consistently high viability on days three and seven, however, the viability of the other three strains decreased steadily. This is in agreement with observations of Krujatz et al., who in a similar approach reported a cell viability of 30% after 6 days of cultivation of bioprinted *C. sorokiniana* under white light and at 37°C (Krujatz et al., 2015). However, the values in the current experiment remain well above the values of Krujatz et al. after 7 days, suggesting that a temperature of 37°C and illumination with 150 µmol/m^2^s of red light seems to be more cell-preserving even under constant illumination and thus more advantageous regarding the viability of the microalgae in the hydrogel.

Comparing the measurements of photosynthetic activity with those of the suspension cultures, *C. striolata* and *C. oocystiformis* appeared to have decreased or even stopped all photosynthetic processes after printing, the cells seemed to starve. This is in accordance with the declining viability for those two strains. *C. sorokiniana* and *Scenedesmus* sp. on the other hand, already reached the values of the suspension culture at day one after printing (Figure 4B). During the printing process, no detrimental stress factors influencing the effectiveness of the photosynthetic processes of *C. sorokiniana* and *Scenedesmus* sp were apparent. This is particularly surprising with respect to *C. sorokiniana*: Whereas the Y(II) values determined for suspension cultures were still in agreement with those measured by Liu t al., the latter observed a halving of the Y(II) to Y(II) = 0.1 when *C. sorokiniana* was immobilized in alginate-based hydrogel beads (Liu et al., 2012). However, the immobilization process of Liu et al. could be considered more stressful for the microalgae due to a longer crosslinking time of 60 min in 200 mM CaCl_2_ crosslinking solution a concentration twice that used in the present study. Higher cell stress resulting from this process may then represent a trigger for the drop in photosynthetic efficiency, as observed by Lui et al. This assumption is also supported by the fact that in that study the cells recovered from day two onwards and reached yield values of up to Y(II) = 0.25 between day three and day six, which again corresponds to the Y(II) values of days three and seven determined in the present study (Figure 4B). The different composition of the hydrogel–sodium alginate used by Liu et al., methylcellulose and sodium alginate in the present work–may also have an influence on the different photosynthetic efficiency at day one: After ionic crosslinking of the alginate, the water-soluble methylcellulose is dissolved over time that results in an increased porosity of the hydrogel as indicated by scanning electron microscopy analysis (Schütz et al., 2017). However, the recovery of the values reported by Liu et al. in the course of the first 2 days rather indicates a direct influence of the immobilization steps than of the hydrogel itself. Embedding in alginate hydrogels is even considered to be particularly well suited for immobilization of microalgae without increasing the stress level of the cells (Moreno-Garrido, 2008). According to Moreno-Garrido, the advantage of alginate-based hydrogels lies in the protection of microalgae from extreme changes in physicochemical environmental conditions both during and after the immobilization process. This is mainly due to the non-toxic effect of the alginate but also due to a shielding effect as the diffusion of substances is retarded in the hydrogel and energy, resulting e.g. from shear forces, is (partially) absorbed by the hydrogel. Furthermore, its transparency allows a sufficient illumination even to microalgae located in deeper regions of the hydrogel (Lode et al., 2015). Hence, comparing the photosynthetic activity in liquid and bioprinted cultures (Figure 4B), *C. sorokiniana* appear to not be influenced by the combination of 37°C and red light after immobilization, as they maintain the same levels of photosynthetic activity throughout the entire cultivation period. The characterization of several green microalgae strands in suspension cultures and in bioprinted constructs identified *Scenedesmus* sp. as the most promising candidate for bioprinted co-cultures with mammalian cells as its viability and photosynthetic activity were maintained under the adapted culture conditions.

Mammalian cells require organic carbon sources in the form of glucose as a key part of their chemoheterotrophic metabolism. Due to the fact that glucose present in the medium can also be consumed by bacteria and mixotrophic microalgae, we furthermore investigated two aspects which we considered highly relevant for the envisaged co-culture: 1) the sensitivity of *Scenedesmus* sp. to antibiotics in the culture medium–antibiotics are commonly added to mammalian cell cultures to prevent bacterial contaminations–and 2) the photosynthetic activity of *Scenedesmus* sp. in the presence of glucose. Most commonly used in mammalian cell culture media to prevent bacterial contamination is a combination of penicillin and streptomycin to inhibit the replication of both Gram-positive and Gram-negative bacteria. The negative effect of streptomycin on growth and photosynthetic efficiency of *Scenedesmus* sp. (Figure 5) is consistent with observations described in the literature, where an inhibiting influence on growth and photosynthetic activity of the microalga *Chlorella vulgaris,* attributed to changes in the algae’s chloroplasts, is described (Perales-Vela et al., 2016). This negative effect might be explained by the mechanism of streptomycin, which is the inhibition of protein biosynthesis by binding to the 30S subunit of the bacterial ribosomes (Pelchovich et al., 2013). The chloroplasts in plant cells and microalgae, which have a bacterial origin, contain a ribosome subunit analogue to the bacterial 30S which may be affected by streptomycin, causing a reduction of the chlorophyll synthesis and hence, an inhibition of photosynthesis. Penicillin’s mechanism of action is based on its interference during the peptidoglycan cell wall synthesis of bacteria by inhibiting the crosslinking stage (Yip and Gerriets, 2022). Since the microalgal cell wall is composed mainly of cellulose, hemicellulose, and pectin, it is not affected by this antibiotic (Blsalputra and Weier, 1963). As an alternative to the penicillin/streptomycin combination that affects Gram-positive as well as Gram-negative bacteria, ampicillin was chosen. Similar to Penicillin, ampicillin inhibits the peptidoglycan crosslinking during cell wall synthesis, which has no effect on microalgae; additionally, it inactivates penicillin binding proteins, part of the resistance mechanism of bacteria (Figure 5) (Peechakara et al., 2021).

The microalgae strain *Scenedesmus* sp. is expected to be mixotrophic and therefore able to consume organic carbon sources like glucose in addition to the photoautotrophic metabolism: it showed higher growth rates and biomass accumulation in a mixotrophic cultivation (Zhao et al., 2012; He et al., 2019). However, the differences in oxygen production rate between photoautotrophic and mixotrophic metabolism were not yet investigated. It was demonstrated here that the selected strand *Scenedesmus* sp. retains a high photoautotrophic portion of its metabolism in the presence of glucose, which will allow the mammalian co-culture partner to proliferate and function unhindered while also reliably providing the necessary oxygen. Figure 7A demonstrates that the behavior of the microalgae is very similar regardless of the glucose content in the media, indicating a very small heterotrophic portion within the algae’s metabolism. The significant decrease in glucose concentration, pictured in Figure 7C, can be explained by the long period in darkness, where the microalgae were able to use the glucose as a nutrition source. This could lead to competition for glucose with the mammalian cells. However, the similar incline of the oxygen concentration in both media shortly after the illumination was restored indicates that the microalgae switch back to a purely phototropic state quickly; Figure 7B illustrates that there is no significant difference in oxygen concentration between both groups. These findings stress the importance of an adequate illumination regime, since longer time spans in darkness can lead to a greater competition for glucose between both cell types.

In comparison, Supplementary Figure S2 shows the oxygen concentration in the supernatant for the strain *C. sorokiniana*, previously used in the short-term co-culture with pancreatic islets reported by Bloch et al. (2006a), Bloch et al. (2006b), under the same co-culture conditions applied for *Scenedesmus* sp. (Figure 7). There, a strong decline in the oxygen concentration in the medium is visible until presumably most of the glucose was metabolized, which suggests a large heterotrophic share in the metabolism and therefore a significantly different behavior of the microalga. Additionally, the glucose content in the media was significantly reduced over the span of 8 hours. It can be hypothesized that different microalgae strains have different grades of mixotrophic behavior: *Scenedesmus* sp. tends to the photoautotrophic side while *C. sorokiniana* possesses a considerably greater heterotrophic portion. The lower values regarding the photosynthetic yield of *C. sorokiniana* might also be explained by this behavior.

In conclusion, there are vast differences between the four investigated microalgae strains in terms of their acceptance of bioprinting and their overall suitability for a possible co-cultivation. The strain *Scenedesmus* sp. CCALA 1074 demonstrated the highest photosynthetic efficiency and viability under the proposed co-culture conditions, making it the most suitable partner for a possible co-culture with mammalian cells. Nevertheless, investigating thermotolerant cyanobacteria in the future could potentially reveal other feasible partners using the same methods proposed here. Embedding both mammalian cells and microalgae in one construct, by precise placement in close vicinity to each other, could provide an oxygen supply for tissue engineered tissues independent from diffusion from air–proving this hypothesis will be the next step in our research.

## Data Availability

The raw data supporting the conclusion of this article will be made available by the authors, without undue reservation.
