# Supplementary material for: Selection of a suitable photosynthetically active microalgae strain for the co-cultivation with mammalian cells
Source: Front Bioeng Biotechnol. 2022 Sep 19;10:994134. doi: 10.3389/fbioe.2022.994134 (PMC9528974; doi:10.3389/fbioe.2022.994134)
Supplement: Supplementary file 1 [file DataSheet1.pdf]

Supplementary Material

1.1 Supplementary Figures

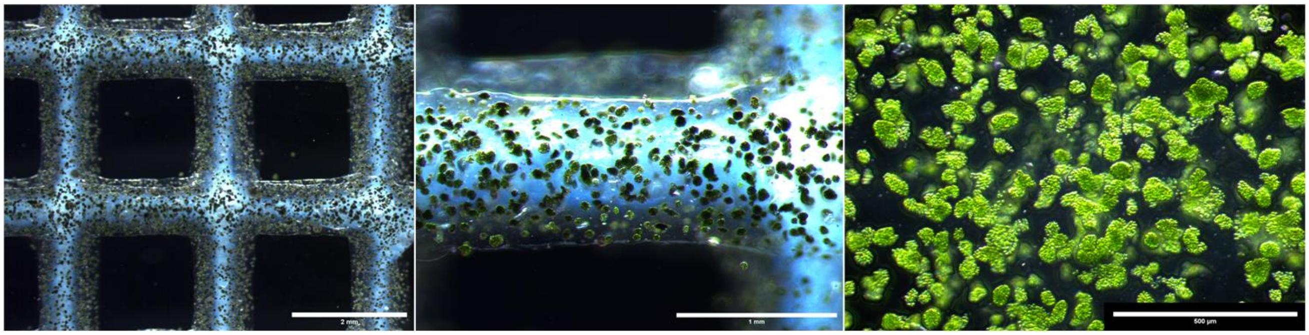

Supplementary Figure 1. Images of bioprinted microalgae under a reflected-light microscope.

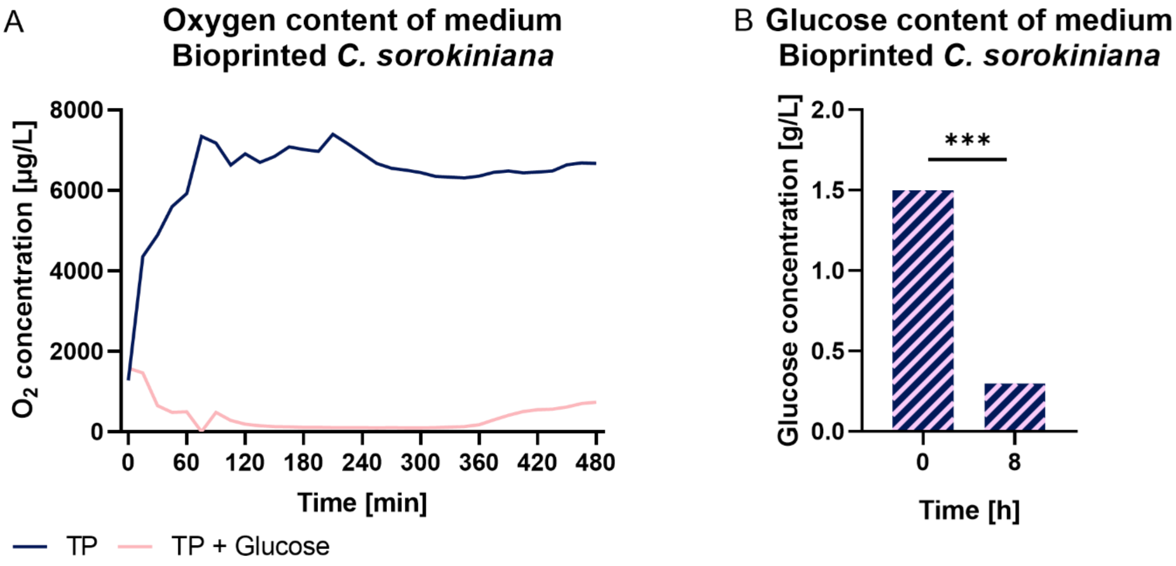

Supplementary Figure 2. Oxygen content over time (A) and glucose concentration (B) of 3D bioprinted *C. sorokiniana*. Depicted are 480 min of cultivation under hypoxic conditions (1 % oxygen concentration) in TP medium with and without 7.1 mM glucose under red illumination (150 µmol/m²s) at 37 °C. Depicted is n = 1 (A), mean ± range for (B) with n = 2; ***p < 0.001.
